# How is the Pharmaceutical Industry Structured to Optimize Pediatric Drug Development? Existing Pediatric Structure Models and Proposed Recommendations for Structural Enhancement

**DOI:** 10.1007/s43441-020-00116-4

**Published:** 2020-02-06

**Authors:** Thomas Severin, Solange Corriol-Rohou, Christina Bucci-Rechtweg, Kristina an Haack, Sabine Fuerst-Recktenwald, Pirkko Lepola, Ensio Norjavaara, Martine Dehlinger-Kremer, the EFGCP Children’s Medicines Working Party, Sebastian Haertter, S. Y. Amy Cheung, the IQ Pediatric Working Group

**Affiliations:** 1 Global Drug Development, Novartis Pharma AG, Novartis Campus, 4002 Basel, Switzerland; 2 Global Regulatory Excellence, AstraZeneca, Paris, France; 3 Global Health Policy, Regulatory Affairs, Novartis Pharmaceuticals Corporation, East Hanover, NJ USA; 4 R&D/Clinical Development Rare Diseases, Sanofi/Genzyme, Chilly-Mazarin, France; 5 Product Development Neurology, F. Hoffmann-La Roche Ltd., Basel, Switzerland; 6 Department of Children and Adolescents, Helsinki University Hospital, Helsinki, Finland; 7 Global Medicines Development, AstraZeneca, Mölndal, Sweden; 8 Pediatric Development, Synteract, & EUCROF, Munich, Germany; 9 Translational Medicine & Clinical Pharmacology, Boehringer-Ingelheim, Ingelheim, Germany; 10 Certara, Princeton, NJ USA

**Keywords:** Medicines for children, Pediatric medicines, Child, Drug development, Pediatric structures, Expert group

## Abstract

**Background:**

Pediatric regulations enacted in both Europe and the USA have disrupted the pharmaceutical industry, challenging business and drug development processes, and organizational structures. Over the last decade, with science and innovation evolving, industry has moved from a reactive to a proactive mode, investing in building appropriate structures and capabilities as part of their business strategy to better tackle the challenges and opportunities of pediatric drug development.

**Methods:**

The EFGCP Children’s Medicines Working Party and the IQ Pediatric working group have joined their efforts to survey their member company representatives to understand how pharmaceutical companies are organized to fulfill their regulatory obligations and optimize their pediatric drug development programs.

**Results:**

Key success factors and recommendations for a fit-for-purpose Pediatric Expert Group (PEG) were identified.

**Conclusion:**

Pediatric structures and expert groups were shown to be important to support optimization of the development of pediatric medicines.

## Introduction

Significant progress has been made since pediatric legislation was first enacted in the USA two decades ago and with the implementation of the Pediatric Regulation in the European Union (EU) in 2007. In regions with pediatric-specific regulations (USA, EU, and Switzerland), it is routine for sponsors to meet with regulatory agencies to discuss pediatric plans for investigational products in their pipelines. In addition, as drug developers have gained pediatric experience and expertise under these regulations, they have started to apply their knowledge to investigational products designed to address diseases primarily observed in pediatric populations. However, key complexities related to pediatric drug development have hindered optimal investment and operational implementation, including the scarcity of available pediatric patient populations, highly competitive therapeutic domains, and minimal financial returns on investment [1], which may be counterbalanced though by the 6-month reward. These complexities have led many sponsors to assess whether existing internal structures and processes tailored to address adult drug development were also fit for purpose to address the unique needs of pediatric drug development. Conducting a comprehensive internal evaluation of current practice in relation to the business needs is one way to assess whether centralizing pediatric expertise will address a drug developer’s organizational needs. To better tackle the challenges and opportunities of pediatric drug development, the pharmaceutical industry has invested in building appropriate structures and capabilities as part of their business strategy, i.e., namely to create a Pediatric Expert Group (PEG). There are two main drivers for a company when establishing a PEG: (1) as first priority, to develop and provide medicines wherever appropriate for pediatric patients to fulfill unmet medical needs and reduce off-label use and (2) to fulfill regulatory requirements. A PEG should not only enhance internal company functioning but also facilitate or establish interactions externally to enhance the quality and efficiency of pediatric medicines development, e.g., through external pediatric research networks, collaboration with academic institutions, public private partnerships (PPP), and patients’ organizations.

As part of their activities, the European Forum for Good Clinical Practice Children’s Medicines Working Party (EFGCP CMWP) surveyed its members to understand how the pharmaceutical industry is organized to support pediatric drug development. While drafting this article, a decision was taken to partner with the Pediatric Working Group/Clinical Pharmacology Leader Group within the International Consortium for Innovation and Quality in Pharmaceutical Industry (the IQ CLPG pediatric working group). The survey method by CMWP utilized open text, while IQ utilized a semi-structured interview. The survey focused on how the PEGs in the CMWP and IQ member companies are structured, their remit, and role and responsibilities to ultimately provide recommendations for others who are considering establishing a PEG. In particular, questions focused on the inter-relation between pediatric and adult development, on whether or not pediatric portfolio management is under the remit of PEG, on the composition of the PEG, and the resources allocated to a PEG. There was good cross-functional pediatric drug development representation since IQ interviewees were mainly clinicians and clinical pharmacologists, and CMWP were clinicians and regulatory personnel. Questions related to budget (e.g., to define whether there are advantages to have pediatric development included in the overall project budget or whether a PEG would have its own budget for pediatric development) were not included in the survey. In parallel to the work done by the CMWP, the IQ pediatric working group had set up interviews with its members between June and November 2018. Both CMWP and IQ answers were then consolidated and used to inform Table 1. No further tool was applied. This article presents a summary of findings from the companies surveyed (Astellas, AstraZeneca, Bayer, Boehringer-Ingelheim, Johnson & Johnson, Novartis, Pfizer, Roche/Genentech, and Sanofi), providing an overview of the variety of PEG structures established to date. Of note, only large and medium pharmaceutical companies were surveyed since there are no small enterprises included in EFGCP and IQ pediatric groups. These findings can serve as recommendations for companies which have interest in establishing a PEG that is fit for purpose.

**Table 1.** Pediatric Expert Group structures in the Pharmaceutical Industry.

Pediatric infrastructure (Model)	Model 1 (Department like)	Model 2 (Medical chair)	Model 3 (Regulatory chair)	Model 4	Model 5
Ownership for the strategic plan of a pediatric program and accountability towards senior management	Pediatric development team (e.g., for specific pediatric indications such as oncology)	Adult project team	Adult project team	Adult project team	Adult project team
Pediatric Strategy across projects tasks^b^/Pediatric Portfolio management	Separate strategic team and portfolio management	Within core expert team	Within core expert team; portfolio management separate	Within core expert team; portfolio management separate	By project teams
Dedicated Pediatric Clinical Expert	Full time^a^	Full time	Full time (some indications part time)	Full Time (some indications part time)	Part time
Regulatory	Full time	Full time	Full time	Full time	Part time
R&D/CMC/Tox	Technical development team lead is part of core expert team	Separate, not part of core	Pharm Dev, DMPK part of core	CMC/Tox	Separate
Clin Pharm, Pharmacometrics and Biostats	Full time (Clin Pharm) in core expert team	Separate, not part of core	Full time in core expert team if focus is on quantitative functions^c^	Part time in core expert team	Part time in core
Other functions	Communication specialist, healthcare, law, finance, real world data, project manager in core team	None	Project manager, patent, and translational science in core expert team	None in core expert team	None in core expert team

^a^Full-time employee: full to ≥ 50%.

^b^E.g., pediatric biomarker; palatability; bio-distribution; extrapolation; adolescents in adult program.

^c^Quantitative functions: Clinical Pharmacology, Pharmacometrics/Modeling & Simulation/Quantitative System Pharmacology, Biostatistics.

This article does not contain any studies with human or animal subjects performed by any of the authors.

## Why is There a Need for a Specific Pediatric Structure?

Integration of pediatric aspects early in drug development has now become normal practice. Pediatric drug development should be driven by the identified pediatric needs and must consider applicable regulatory requirements and recommendations [2]. With the implementation of several pediatric policies in both Europe and the USA, and the strong support of various advocacy groups and of the pediatric community, pediatric research has made significant progress [3, 4] over the last decade. To make safe and effective medicines available for the pediatric population, timely development of evidence on the proper use of products in pediatric patients of various ages is needed, taking into account not only regulatory guidelines [2] and clinical recommendations, but also specific tools, trial designs, and methods [5], including the pharmaceutical design of pediatric formulations [6]. When developing medicines for pediatric patients, it is essential to identify the patient population and its specific characteristics and medical needs, the environment where the medicine is likely to be used (e.g., hospital or community), the existing knowledge, and the medicine’s acceptability/palatability [7–9]. Preliminary feasibility assessment of pediatric research networks conducted at an early stage in the pediatric development program, a key prerequisite to optimize trial operations and execution, should systematically involve pediatric patients’ organizations and/or their caregivers [10, 11]. Their contribution should lend credible and realistic insights regarding the feasibility of clinical trial conduct.

## What are the Current Existing Models of Pediatric Expert Group Structures in Industry?

An important finding of the surveys is that numerous different types of PEG infrastructures can support pediatric drug development. While structures differ, all companies surveyed have acknowledged that the obligation to deliver pediatric drug development programs, to address specific pediatric medical needs, and to comply with the fast-evolving regulatory environment play an important role when establishing a PEG. While the companies surveyed have established internal PEGs, the structures, roles and responsibilities, and scope of work deviate substantially from company to company. The range of structures spans from a looser aggregation of subject matter experts who meet on an ad hoc basis to pediatric-specific departments with governance function. Accordingly, the mandate varies substantially, with some PEG having consultative capacity only (see Model 5 in Table 1), while others have strategic ownership and full accountability for the pediatric development program (see Model 1 in Table 1).

While there are varied responsibilities of PEGs, there appears to be a consistent set of core functions that contribute to the PEG regardless of their structural make-up across companies. Besides medical (i.e., pediatricians) and regulatory expertise, clinical operations and clinical pharmacology/pharmacometrics are usually represented in these PEGs.

A factor determining the size, mandate, and diversity of any PEG appears to relate to the company’s portfolio. A strong focus on oncology within the regulated pharmaceutical industry may make certain models more efficient in addressing such therapeutic needs. In particular, while the European Medicines Agency (EMA) considers the broader condition to require pediatric cancer drug development, the U.S. Food and Drug Administration (FDA) will specifically require pediatric assessment in all New Drug Applications and Biologics License Applications submitted for adult oncology indication after 19 August 2020, in cases where the molecular target of the candidate molecule is substantially relevant to cancers in childhood [12]. In such cases, a structure where the PEG is responsible for the program strategy may be reasonable and useful (see Model 1 in Table 1).

For many companies, pediatric development is part of the overall product strategy driven by the adult project teams, and the key mandates of PEGs are a provision of their specific expert consultative advice on all aspects of pediatric development. This collaboration can be fostered with key external academic experts, pediatric research networks, advice from regulatory bodies, and/or external pediatric initiatives that serve to keep the PEG infrastructure up to date.

Common to all PEG structures, is a strong focus on medical functions, with expert pediatricians usually assigned per therapeutic area. For example, the Johnson & Johnson’s ‘Child Health Innovation Leadership Department (CHILD)’ is a core pediatric department formed almost exclusively by pediatricians to guide the company’s pediatric portfolio (see Model 2 in Table 1). Besides the medical knowledge and expertise in pediatric indications, pediatricians may act as key contact to principal investigators, external clinical experts, pediatric hospitals, and medical societies, as well as pediatric research networks, and may be supported by other functions where needed, e.g., when it relates to policy or quantitative issues.

An iteration from the medical/pediatrician-led model is one including a regulatory expert as the chair of the PEG (see Model 3 in Table 1). However, when the regulatory expert is not the chair often multiple regulatory experts are part of the PEG. Across the industry, there is no consistency in the given role of regulatory experts within the PEGs. Some may hold responsibility only for the pediatric strategy and interactions with the regulatory authorities, while in other PEGs, regulatory experts are actively engaged in shaping regulatory policy both specific to pediatrics and to other crucial development topics including but not limited to access, pricing and reimbursement, or innovative analytics.

Expansion of the PEG by further functions such as clinical pharmacologist, toxicologist, pharmaceutical development (CMC), safety or legal experts, epidemiologist, as well as biostatistician ranges from full to almost full-time engagement in addition to routine project work, in order to deliver expertise for innovative study designs or to support extrapolation efforts. Pharmacometrician involvement in the PEG seems to be common practice to provide expertise in quantitative clinical pharmacology, clinical and nonclinical Modeling & Simulation (M&S), bioinformatics, Health Technology Assessment, and payer's evidence as well as in translational science.

All companies participating in the survey advised that clinical operations is an essential part of the PEG although the number of clinical operation experts and their level of dedication differed in whether they only support pediatric studies or are also responsible for adult clinical development programs.

While some PEGs include pediatric safety experts, others rely on the expertise provided by the project team or included in specific safety knowledge groups, e.g., to provide expertise on the impact on the liver, cardiac, or immune system.

Quite often, nonclinical and CMC functions are included in the core PEG although they may serve as part of a separate expert group, e.g., a nonclinical PEG, and functioning mostly through their respective project teams.

Epidemiology, real world data, and legal advice/patent experts can either be included in the core of a PEG or consulted on an ad hoc basis.

When the responsibility for the strategic pediatric drug development plan remains with the adult project team, a common feature for most companies surveyed, some differences were noted in the use of PEG for counsel and advice. For most of the surveyed companies, it is mandatory to reach out to the PEGs early for discussion on the pediatric drug development strategy and review of regulatory documents such as Pediatric Investigation Plan (PIP), Pediatric Study Plan (PSP), or clinical study protocols (CSP); for others, such an approach is voluntarily. Similarly, in most companies surveyed PEGs are used to develop internal guidance documents, best practice standards, and template documents and to provide regulatory intelligence expertise to help project teams designing their pediatric development program. Often, specific internal guidance documents (e.g., CMC, clinical pharmacology or M&S) are developed not only to ensure efficiency of process, but also to build functional capability and facilitate knowledge transfer.

Additionally, many companies noted that the point of contact for a specific function often leads a broader pediatric-focused subject matter expert team (e.g., formulation expert team or clinical pharmacology expert team) that can be pulled in to address specific issues that may arise.

### Simplifying the 5 Models of the Pediatric Group Structures

Based on companies’ feedback received by both the EFGCP CMWP and the IQ consortium, five different models of PEG structures were identified (Table 1) which can be easily re-grouped into three distinct general types of models:

1. Informal advisory pediatric networks: this type of network requires a person who is dedicated to pediatric drug development and can identify other subject matter experts with various expertise, e.g., CMC, Nonclinical, or Technical Development. In these PEGs, member involvement depends on the time allocated to provide support and advice on pediatric programs. When pediatric strategy and programs are developed, it is common practice to create a pediatric drug development team with various persons from different functions. These persons can come from the informal PEG and vice versa and can become a PEG member as they gain pediatric experience (Model 5 of Table 1).
2. Formal advisory pediatric networks: this type of network requires subject matter experts working part or full time across all functions, which increases the quality of the scientific support provided. In this model, the individual project teams retain responsibility for the product’s strategic plan, whereas the PEG serves to provide consultation on pediatric-specific components to the product plan. Dependent on the company, these formal advisory networks may also provide a pediatric governance function, e.g., for the review of PIP, PSP or/and CSPs (Models 2, 3, and 4 of Table 1).
3. Pediatric departments/team with the responsibility for pediatric programs: in this situation, pediatric knowledge is consolidated and centralized within the company into one department with dedicated personnel from all contributing functions. Such a model (Model 1 of Table 1) allows for a pediatric portfolio-based development paradigm which manages the pediatric strategy separately from the overarching product strategy.

## How to Define the Most Appropriate Fit-for-Purpose Pediatric Structure?

Defining the most appropriate pediatric structure for a company can only be done after an analysis of the potential efficiency and productivity benefits each model could offer, in line with pipeline opportunities, and the existing scientific and regulatory environment.

### Analysis of Efficiency and Productivity

When conducting a business review to assess the potential efficiency and productivity benefits for establishing a PEG in relation to pediatric program development needs, careful efforts should be undertaken to comprehensively analyze internal metrics, from discovery to product registration and beyond authorization. These may include but are not limited to therapeutic areas of interest and related modes of action and analysis of timelines: from discovery to entry into the clinic for the first in pediatric (FIP) patient; FIP to first pediatric marketing authorization; number of regulatory interactions with the same health authority (e.g., EMA) to agree on a pediatric program design; number of regulatory interactions with the same regulatory authority (e.g., EMA) to modify an agreed pediatric plan; and the rate of approval on first cycle submission for pediatrics. There should also be the need to look at pediatric-specific trial costs, including but not limited to cost per patient in relation to adult studies; internal and external research organization (e.g., CRO) costs in relation to adult studies; and pharmaceutical development costs for age-appropriate formulations and their maintenance in the market post authorization. These pediatric development costs should all be benchmarked internally between pediatric projects first (some may have benefited from the availability of experts such as pediatricians in the project team or with external consultation) or against competitors who have ongoing development in the same therapeutic area and patient populations. Providing a clear picture of the existing efficiency and productivity (or lack thereof) related to the delivery of pediatric program development is considered essential to understand whether specific PEG structures may be needed and in which format. As contributors to this survey were all from medium to large size pharmaceutical companies, it may be difficult to infer from the survey results any recommendation for small size companies. If small size companies plan to include pediatrics in their drug development program, they may either develop the program on their own, rely on external consultants or on contract research organizations (CROs) with a pediatric center of excellence, or defer the start of their pediatric program for strategic development to future out-licensing partners.

### Analysis of Pipeline Opportunity

Targeted therapies research is rapidly revolutionizing how treatments for the most complex diseases can be developed, which has the potential to facilitate innovation targeted directly at pediatric diseases [13]. As precision medicine and tissue agnostic development approaches continue to emerge as key pipeline drivers, and with increase in disease pathophysiology knowledge, more opportunities may emerge in early phase development for investment in innovation for rare or ultra-rare pediatric diseases. To accommodate this movement, companies need to ensure that they have considered its impact on the need for more extensive pediatric-specific expertise in basic science research and early phase translational development [14]. In addition, as global markets continue to shift and impact on investment in innovation, it is becoming increasingly important for a pediatric business case to consider common pediatric morbid-mortality in the third world e.g., with neonatal sepsis, childhood respiratory infections, diarrhea, malnutrition, or parasitic infection [15]. To be prepared for these new opportunities, a pediatric structure may be considered as a prerequisite to accommodate any new demands.

### Analysis of the Scientific and Regulatory Environment

A good understanding of the environment and its opportunities can help address some of the well-known pediatric drug development challenges.

Collaboration with pediatric research networks can help overcome study feasibility or patient recruitment challenges by providing appropriate solutions. Pediatric research networks can also provide useful insights into national and local or legal and administrative requirements and can promote collaboration between other stakeholders like local regulatory authorities or patient groups. For these reasons, as per Article 44 of the EU Pediatric Regulation [16], the EMA had to develop, with the scientific support of its Pediatric Committee (PDCO), a European network of existing national and European networks, investigators, and centers with specific expertise in the performance of studies in the pediatric population. The European Network of Pediatric Research at the EMA [17] (Enpr-EMA) which started its operations in May 2010 currently includes 52 research network members with a variety of structures and levels of activity [18]; national and European disease-specific, age-specific, or multispecialty networks, centers, and investigators with expertise in pediatrics and performing clinical studies in children. Over these last 10 years, the network has grown substantially and has even expanded beyond Europe with networks in the USA, Canada, and Japan [19]. All of these networks represent a vast source of expertise [20] although most of them are still underutilized. A survey [21] carried out by the Enpr-EMA Working Group of PPPs in 2014 to increase network visibility and understand industry expectations of networks resulted in 70 responses from a variety of companies, including large pharma, biotech, consultants, and Contract Research Organizations (CROs). A majority of respondents (66%) was already aware of Enpr-EMA although only 46% had already worked with Enpr-EMA or another research network. A large proportion (71%) of the respondents were from companies with a PEG, which may be seen as evidence that the collaboration with academic networks benefits from stable contacts within industry which can only be guaranteed when a pediatric structure exists. Generally, the respondents highlighted that collaboration with research networks is very valuable and beneficial in providing different services to the pharmaceutical industry. The need for improvement was identified though to address the lack of consistency and uniformity in the network services; something Enpr-EMA has planned to focus on in the coming years. Since pediatric programs are by essence global, it is important to also consider existing ex-EU research networks, such as the International Neonatal Consortium [22].

During recent years, there has been very meaningful improvements in clinical research, and in particular with patient engagement or public involvement. It is now widely accepted that stakeholders’ involvement in clinical research may have a significant impact on clinical trial conduct, resulting in more successful patient enrolment and retention [23]. An important evolution is that researchers and companies see the value and benefits of involving children, young people, and their families as partners in the design and delivery of clinical trials. Involving representatives of the targeted patient populations in clinical research has many interesting benefits, including greater understanding of young people’s perspectives and disease-specific details and improvements in the study design and the quality of the research.

In May 2017, the European Young Person’s Advisory Group network [24] (eYPAGnet) was launched with the aim of empowering young people and families across Europe to contribute to pediatric health research. Currently, the eYPAGnet provides a single, centralized point of contact for the collaboration of the Young Person’s Advisory Group (YPAG) with the relevant working parties of the EMA, including Enpr-EMA and the PDCO, and with European Initiatives including the IMI conect4children (c4c) consortium or the Pediatric Clinical Research Infrastructure Network (PEDCRIN) [25, 26]. Involving young people in the drug development process from clinical trial design through to trial delivery (e.g., trial procedures, outcome measure selection, review of informed consent form or of patients’ diaries) has been seen to be of value.

Finally, through the assessment of pediatric multi-stakeholder initiatives by a company, PEGs have been shown to provide value to project teams, since they offer an opportunity to strengthen clinical research and develop solutions for important issues. The EU Innovative Medicines Initiative [27] (IMI) is a good example with its PPP projects aimed at accelerating the medicines development process, generating new scientific insights, and developing resources for open use by the research community. With the IMI c4c consortium [28] launched in 2018, the expectation is to create a sustainable, integrated, pan-European collaborative pediatric network to speed up and facilitate the running of high-quality clinical trials in children while ensuring that the voices of young patients and their families are heard. This six-year project is one of the biggest initiatives funded by the Innovative Medicines Initiative 2 Joint Undertaking (IMI2 JU).

## Fit-for-Purpose Pediatric Structure

Companies, which have undertaken a detailed evaluation of efficiencies and productivity related to current pediatric drug development that is responsive to regional regulatory requirements (e.g., EU Pediatric Regulation (EC) 1901/2006, U.S. PREA) and have evaluated pipeline opportunities, can start mapping out appropriate options to address their organization’s pediatric drug development needs. In building the pediatric business case to obtain the company’s management support, it is important to construct a plan that reflects each company’s organizational culture as it is likely to inform the type and nature of pediatric expertise that will be best suited to influence pediatric-focused organizational changes. Therefore, we acknowledge that no one type of PEG may work for all companies. However, based on experience, it is strongly suggested to carefully consider a few key success factors (Table 2) to ensure that the pediatric structure is designed to meet organizational needs and support the integration of pediatric aspects early in drug development; in other words, a structure that is fit for purpose.

**Table 2.** Key Success Factors: Questions to Address for a Fit-for-Purpose PEG.

1. What organizational culture change is required for the PEG and the utility it may offer to be “bought into”? Is organizational information, advocacy, and training on pediatric needs required?	
2. Is there a champion within the company’s management structure? Are they engaged and successful as an influencer?	
3. What is the mandate for the individual PEG?	
– Delivering on efficiency in regional regulatory requirements? < – > Driving innovation for pediatric drug development?	
– Is the mandate region specific or is it intended to address global pediatric drug development needs?	
4. What function and role will the PEG serve organizationally?	
– Internal consultancy to operationalize pediatric program development?	
– Will the PEG serve any governance function (e.g., sign off on strategic planning or protocol review)?	
5. What composition is required to deliver on each organizational mandate? Clinical and clinical operations only or cross-functional program strategy including e.g., discovery and technical development?	
6. Are there critical foundational start-up activities that are required (e.g., pediatric data standards, pediatric assent templates, pediatric protocol templates) before the PEG can focus its deliverables on the organizational mandate?	
7. What is the resource commitment (personnel and financial) that is needed to credibly deliver the organizational mandate?	
8. What are the deliverables the PEG can credibly deliver to the organization in 1 year, 3 years, 5 years, or 10 years?	
9. What is the estimated impact organizationally (e.g., efficiency, quality) and societally?	
10. Is there senior management support?	

It is important to establish a cross-functional group for project team support and external network interface with a group Chair, at least partially dedicated to pediatrics. Chairs, who are not dedicated full time, should be assigned a sufficient portion of their overall time (at least 20%) to ensure that the objectives of the PEG can be executed while meeting the needs of the organization. The PEG should include representatives of the core functions (e.g., pediatricians, regulatory, clinical pharmacology, pre-clinical, CMC, patient safety, clinical operations experts) for whom their pediatric work should be a part of their individual objectives. Such a group needs to support internal initiatives such as training seminars, shared learning sessions, “best practice” documents (e.g., pediatrics vital signs, ECG thresholds, blood sampling and contraception guidelines, or consent/assent templates), and selected external pediatric activities (e.g., involvement in pediatric conferences or in PPPs). Finally, the PEG needs to have senior management endorsement demonstrating a commitment within the company mindset to support pediatric plan development—not only as an obligation but also as an opportunity. In that context, it should be discussed how the adult program could be utilized to better inform the pediatric strategy. This could be done to support the development of an extrapolation concept, of an additional biomarker, or of pediatric clinical endpoints prior to their incorporation into the pediatric program. A PEG is an excellent expert resource for adult development teams to seek guidance when considering these pediatric needs early in a drug development program.

When building a PEG, defining a work plan with annual objectives and achievements, resource demands, as well as means for regular communication through Town Hall meetings or via a dedicated share point site is important to demonstrate the PEG’s utility.

## Conclusion

More than 10 years after the implementation of the Pediatric Regulation in Europe, it was interesting to survey the pharmaceutical industry to understand how companies are organized to tackle the challenges and opportunities of pediatric clinical research. All respondents were representatives of large and medium pharmaceutical companies which have PEGs in place, but whose structures differ from one company to the other. All respondents agreed that it is of utmost importance to protect the rights of pediatric study participants, to optimize pediatric studies, to minimize the exposure of children to unnecessary clinical studies, and ultimately to increase the number of approved medicines for children. Based on this experience, we issue these recommendations for companies that may need such a structure, highlighting that a key to success is a fit-for-purpose PEG. Such a structure may help to optimize company’s processes already in place to better address the development of pediatric medicines and a rapidly evolving regulatory and scientific framework.
